# Recent transfer of an iron-regulated gene from the plastid to the nuclear genome in an oceanic diatom adapted to chronic iron limitation

**DOI:** 10.1186/1471-2164-11-718

**Published:** 2010-12-20

**Authors:** Markus Lommer, Alexandra-Sophie Roy, Markus Schilhabel, Stefan Schreiber, Philip Rosenstiel, Julie LaRoche

**Affiliations:** 1 Leibniz Institute of Marine Sciences at Kiel University IFM-GEOMAR, Kiel, Germany; 2 Institute of Clinical Molecular Biology, Christian-Albrechts-University Kiel, Kiel, Germany

## Abstract

**Background:**

Although the importance and widespread occurrence of iron limitation in the contemporary ocean is well documented, we still know relatively little about genetic adaptation of phytoplankton to these environments. Compared to its coastal relative *Thalassiosira pseudonana*, the oceanic diatom *Thalassiosira oceanica *is highly tolerant to iron limitation. The adaptation to low-iron conditions in *T. oceanica *has been attributed to a decrease in the photosynthetic components that are rich in iron. Genomic information on *T. oceanica *may shed light on the genetic basis of the physiological differences between the two species.

**Results:**

The complete 141790 bp sequence of the *T. oceanica *chloroplast genome [GenBank: GU323224], assembled from massively parallel pyrosequencing (454) shotgun reads, revealed that the *petF *gene encoding for ferredoxin, which is localized in the chloroplast genome in *T. pseudonana *and other diatoms, has been transferred to the nucleus in *T. oceanica*. The iron-sulfur protein ferredoxin, a key element of the chloroplast electron transport chain, can be replaced by the iron-free flavodoxin under iron-limited growth conditions thereby contributing to a reduction in the cellular iron requirements. From a comparison to the genomic context of the *T. pseudonana petF *gene, the *T. oceanica *ortholog can be traced back to its chloroplast origin. The coding potential of the *T. oceanica *chloroplast genome is comparable to that of *T. pseudonana *and *Phaeodactylum tricornutum*, though a novel expressed ORF appears in the genomic region that has been subjected to rearrangements linked to the *petF *gene transfer event.

**Conclusions:**

The transfer of the *petF *from the cp to the nuclear genome in *T. oceanica *represents a major difference between the two closely related species. The ability of *T. oceanica *to tolerate iron limitation suggests that the transfer of *petF *from the chloroplast to the nuclear genome might have contributed to the ecological success of this species.

## Background

In contemporary oceans, diatoms account for approximately 40% of the oceanic primary production and play a critical role in the sequestration of atmospheric CO_2 _into the deep ocean [1]. The high diversity of diatoms and their cosmopolitan distribution in the marine environment reflect the ecological success endured by this group since their first appearance more than 150 Ma ago [2].

Although diatoms thrive in coastal areas where dissolved nutrients are high, many species of diatoms are prevalent in high-nutrient, low-chlorophyll (HNLC) oceanic regions where primary production is chronically iron-limited [3]. Iron fertilization experiments in HNLC regions have repeatedly demonstrated the ability of opportunistic diatom species to bloom once iron is no longer growth limiting [4]. In contrast, some diatom species such as *Thalassiosira oceanica *thrive equally well in the presence or absence of iron [5]. A key determinant for the survival and growth of phytoplankton under iron limitation must be the ability to carry out photosynthesis efficiently, despite the high iron requirements of the photosynthetic infrastructure.

The photosynthetic apparatus, largely contained in the chloroplasts, is jointly coordinated by the plastid and nuclear genomes, involving more than 700 genes [6]. The chloroplast genome of most species generally retained less than 200 of the genes contributing to chloroplast function, as the majority of the endosymbiont's chloroplast genes have been lost or incorporated into the host nuclear genome. The plastids of diatoms and other chromalveolates, originated from a secondary endosymbiosis with a red alga, have retained a higher proportion of the symbiont's genes in their genomes relative to their green counterparts, which derived from a primary endosymbiosis with a cyanobacterium. The red origin of the chloroplasts [7] and the lower cellular iron requirements of the red lineage [8] may have contributed to the ecological success of diatoms in the marine environment in terms of a putative evolutionary-based pre-adjustment to iron-deplete conditions.

The retention of a core set of chloroplast genes and the factors preventing their transfer to the nucleus are the subject of ongoing debates [9]. The known chloroplast genomes of diatoms are circular with an extended inverted repeat region (IR) and are subject to internal rearrangements such as inversions [10]. Occasional organelle lysis and free release of organellar DNA is considered an important first step in the transfer of organelle-encoded genes to the nuclear genome. Indeed high quantities of chloroplast (cp) and mitochondrial (mt) DNA are frequently transferred and inserted into the nuclear genome, thereafter referred to as nuclear plastid or nuclear mitochondrial DNA (NUPTs, NUMTs) [11-14]. However, stable replacement of a plastid gene by its nuclear copy requires a retargeting of the nuclear gene product back to the chloroplast compartment as well as a functional expression and regulation. Until this multi-step development has been accomplished the chloroplast version cannot be discarded and the gene exists in two copies, which might even overlap in function in a differentially regulated manner [15]. Genes that provide a dual targeting sequence enabling import to both mitochondria and chloroplasts at the same time are also known [16].

Here, we present the complete *T. oceanica *CCMP1005 chloroplast genome sequence [GenBank: GU323224], which we assembled from a massively parallel pyrosequencing data set. Assembled genomic shotgun reads show that in the *T. oceanica *genome, the ferredoxin *petF *gene has been transferred to the nucleus. Ferredoxin is a photosynthetic redox protein that contains iron, and in diatoms it can be replaced by the iron-free flavodoxin, when iron-limited growth conditions prevail. The *petF *transfer to the nuclear genome may enable a refined regulation of this gene in response to iron availability in *T. oceanica*. Through comparative genomics between the coastal *Thalassiosira pseudonana *and the oceanic *T. oceanica*, we can trace the *T. oceanica PETF *gene back to its chloroplast origin and identify elements that may have played a role in the transfer of this important photosynthetic gene.

## Results

### Characteristics of the *T. oceanica *chloroplast genome

**Figure 1:**
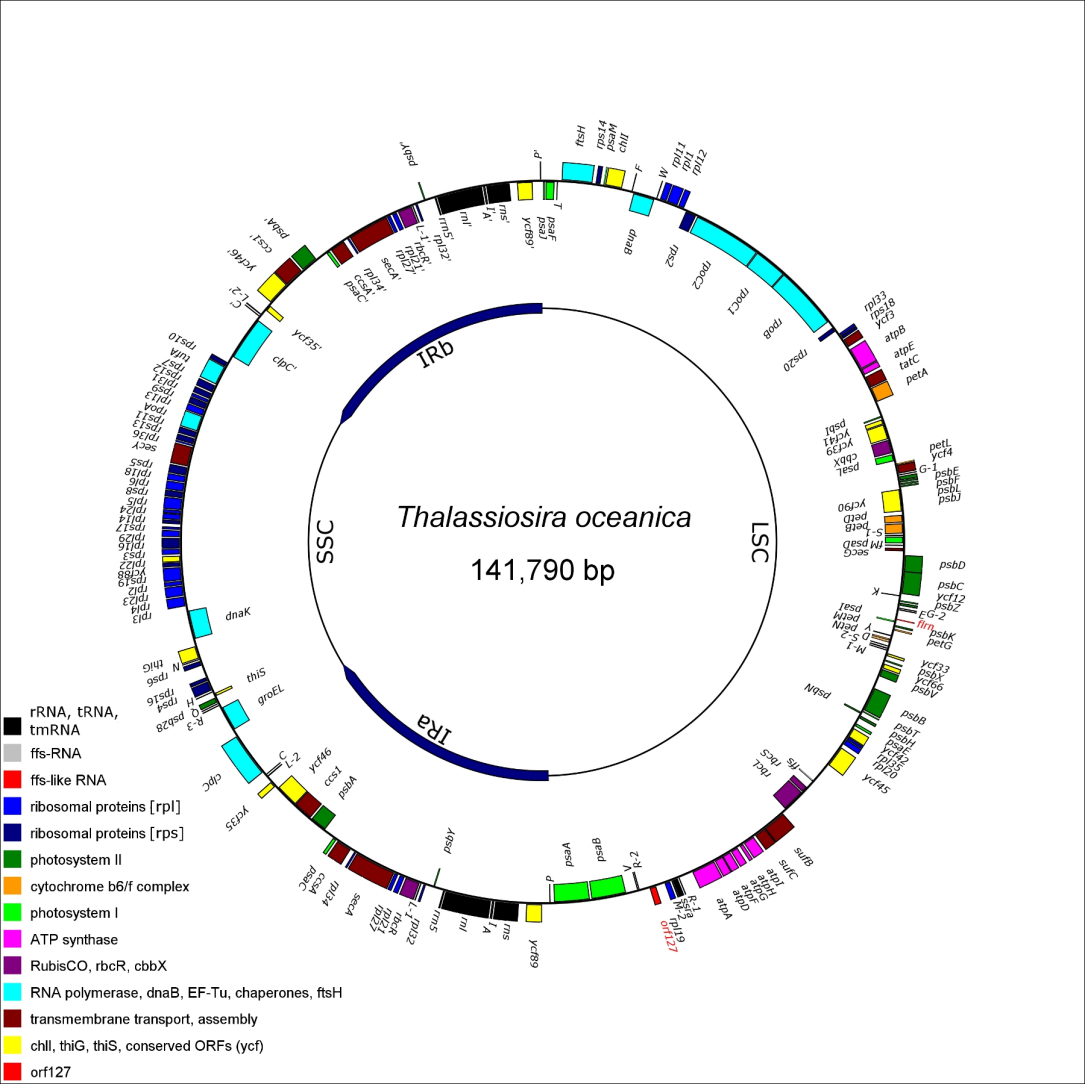
**Circular Map of the *Thalassiosira oceanica *CCMP1005 Chloroplast Genome**. Genes on the outer *forward *strand are transcribed clockwise, genes on the inner *reverse *strand counterclockwise. Gene symbols are colour-coded according to functional groups as indicated, tRNA genes are represented by single-letter code. Features not present in *T. pseudonana *CCMP 1335 are marked red (***orf127***, ***flrn***). **LSC **large single-copy region; **SSC **small single-copy region; **IRa **and **IRb **inverted repeat regions

The cp genome of *T. oceanica *has a physical size of 141790 bp and maps to a circular topology (Figure 1), slightly larger in size than the published cp genomes of the closely related *T. pseudonana *and the more distantly related *P. tricornutum *(Table 1). The cp genome of *P. tricornutum *shows a significantly higher G+C content and contains three protein coding genes not present in the cp genomes of *T. oceanica *or *T. pseudonana*, while the 6.9 Kbp inverted repeat region is significantly smaller than in the two *Thalassiosira *species. The gene composition of the large and small single copy regions (LSC, SSC) and inverted repeat subdomains (IRa, IRb) is nearly identical between *T. oceanica *and *T. pseudonana*. The differences in the overall size of their cp genomes can be solely attributed to the expansion of the inverted repeat region in *T. oceanica *leading to a *de facto *duplication of the three genes *clpC*, *trnC *and *trnL*, and to the loss of the *petF *gene from the large single copy region. Novel features of the *T. oceanica *cp genome are the appearance of an expressed *orf127 *at the site affected by the *petF *gene transfer event, and the partial duplication of the RNA gene *ffs *referred to as *flrn *(ffs-like RNA). Synteny between *T. oceanica *and *T. pseudonana *is weakly conserved (data not shown) and indicates a high degree of dynamic restructuring of chloroplast genomes mainly in form of small scale inversions, though restricted to the respective subdomains (LSC, SSC, IR) without crossing the borders of these elements (compare [10]).

**Table 1:** Chloroplast Genome Features of *T. oceanica* in Comparison with *T. pseudonana* and *P. tricornutum*

	*Thalassiosira oceanica *CCMP 1005	*Thalassiosira pseudonana *CCMP 1335	*Phaeodactylum tricornutum *CCAP 1055/1
**genome size [bp]**	**141 790**	**128 814**	**117 369**
LSC	70 298	65 250	63 674
SSC	24 106	26 889	39 871
IR	**23 693**	IRa: **18 338**	**6 912**
		IRb: 18 337	

**G+C content [%]**	**30,39**	**30,66**	**32,56**

**A+T content [%]**	69,61	69,34	67,44

**gene content [#]**	**158+2**	**159**	**162**
protein coding	126 +*orf127*	127	130
rRNA	3	3	3
tRNA	27	27	27
other RNAs	2 +*flrn*	2	2

in ***T. oceanica***, but not in *T. pseudonana*	***orf127*^a^**, *flrn*		

in ***T. pseudonana***, but not in *T. oceanica*		***petF*^a^**	

in **IR of *T. oceanica***, but not in IR of *T. pseudonana*	***clpC*, *trnC*, *trnL***		

in ***P. tricornutum ***only			***tsf, syfB, acpP***

**^a ^**presence or absence of ***petF ***and ***orf127 ***in *T. oceanica *is the result of a gene transfer event

Genes duplicated in the IR are counted once. The increased genome size of *T. oceanica *can conveniently be explained by an expansion of the IR region. ***Orf127 ***and ***flrn ***are potential pseudogenes and are listed as additional gene content (+2).

### Transfer of *petF *gene to the nuclear genome in *T. oceanica*

**Figure 2:**
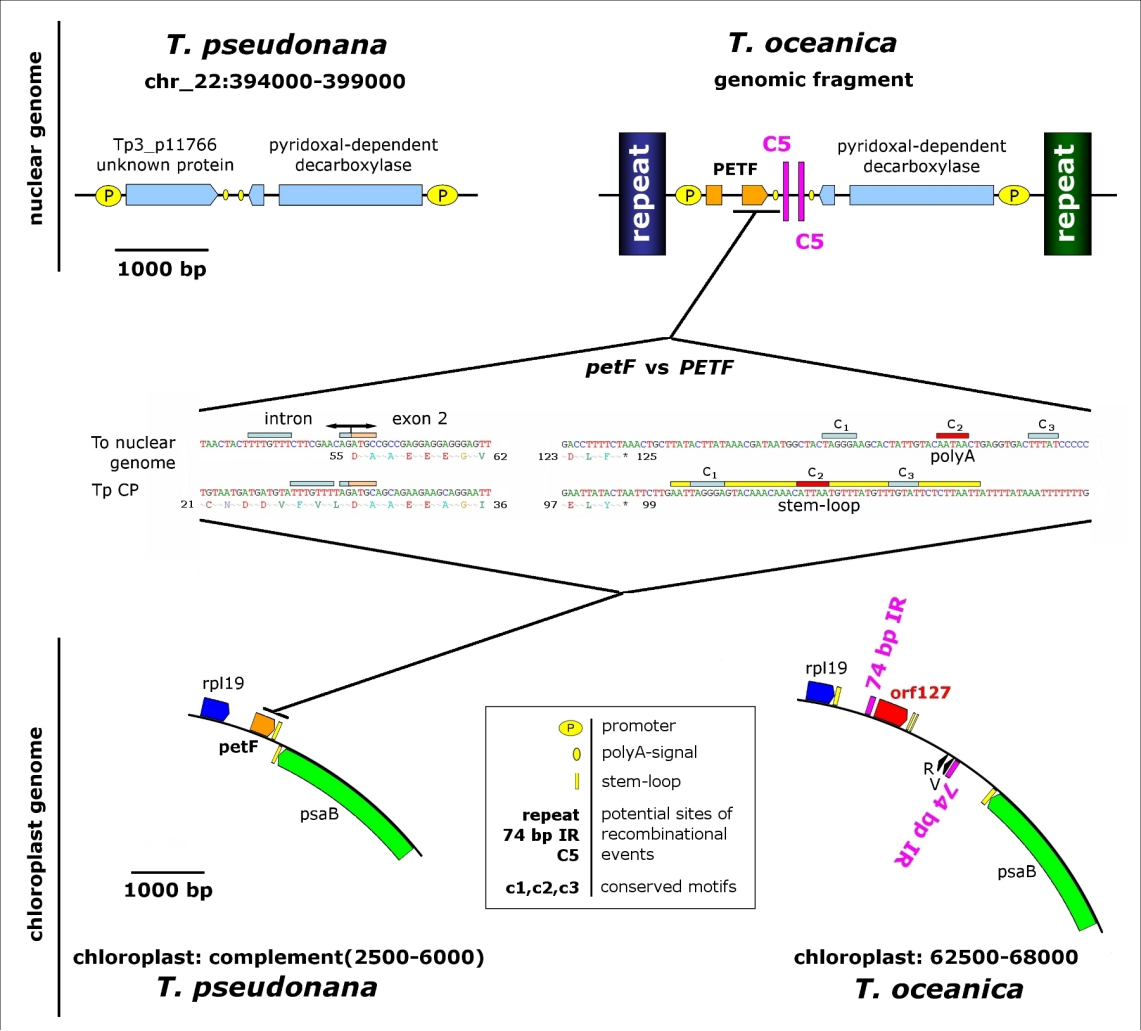
**Genomic Context of the *Thalassiosira petF *Gene Transfer**. The genomic context of the ferredoxin genes in *T. oceanica *and *T. pseudonana *indicates a recent gene transfer event. The *T. pseudonana ****petF ***gene is part of the cp genome and is located between ***rpl19 ***and ***psaB ***(bottom left), serving as a model for the genomic situation in a common ancestor of the two species. The *T. oceanica ****PETF ***gene is found in the nuclear genome next to a *PDD *gene encoding for a pyridoxal-dependent decarboxylase (upper right) with a conserved ortholog present in *T. pseudonana *(upper left). The sites of excision (chloroplast) and insertion (nuclear fragment) in *T. oceanica *show major genome rearrangements in conjunction with the presence of a novel protein coding gene ***orf127 ***in the chloroplast. Both nuclear and chloroplast sites in *T. oceanica *contain various forms of repeat elements („repeat", „74 bp IR") and motifs („C5") that point to the recombinational events underlying the complex multi-step gene transfer (upper and bottom right); these are not present at the comparable sites in *T. pseudonana*. An alignment between the *T. pseudonana *and *T. oceanica *ferredoxin genes reveals sequence similarities at what might be the 5' and 3' ends of the transferred DNA segment (center). The *T. pseudonana ****petF ***coding sequence already contains a potential functional splice acceptor site near the N-terminus of the reading frame. A comparable situation in the ancestral *T. oceanica *cp genome would have facilitated the acquisition of an intron in a new genomic context leaving the major conserved part of the protein unaffected by the process (center left); the stem-loop (yellow bar), that serves as a transcriptional terminator for ***petF ***in the *T. pseudonana *cp genome, contains motifs that are retained in the nuclear genomic context of *T. oceanica ****PETF***, one of them being slightly modified to become an eukaryotic polyA-signal (center right).

The ferredoxin *petF *gene is not present in the cp genome of *T. oceanica *indicating a loss or transfer of that gene to the nucleus. To address this question, BLAST analysis of 454 whole genome shotgun data was performed and revealed the presence of a nuclear genomic contig containing a complete *PETF *gene, confirming the gene transfer event. The nuclear genomic context of the *PETF *region could be assembled manually from the 454 sequence read data and was compared to the syntenic region of *T. pseudonana *(Figure 2). In the *T. pseudonana *cp genome, the *petF *gene is located between the genes *rpl19 *and *psaB*; the comparable site in *T. oceanica *shows the *T. pseudonana petF *gene being replaced by a sequence block consisting of the novel *orf127 *and the tRNA genes *trnV *and *trnR*. Interestingly, the latter unit is surrounded by an unusually large dispersed 74 bp inverted repeat, indicating major genomic rearrangements in that region mediated by these elements and involving a recruitment of the two tRNA genes from a more distant site. Mapping of the *PETF *gene on the nuclear genomic fragment revealed a *PETF *gene model composed of two exons separated by an intron. The first exon encodes a chloroplast targeting peptide, while the second exon carries the information for the conserved functional part of the ferredoxin gene product. The predicted gene model reveals a nuclear promoter as well as a polyA-signal. The *PETF *gene and the adjacent *PDD *gene encoding for a pyridoxal-dependent decarboxylase [17] are surrounded by larger non-related repeat elements (>1000 bp) likely representing remnants of recombinational genomic rearrangements involved in the *petF *gene transfer event. A highly conserved ortholog of the *PDD *gene exists in *T. pseudonana *and its genomic context is shown as a virtual destination for the gene transfer in *T. oceanica*. The chloroplast's contribution to the newly formed nuclear *PETF *gene must at least be the conserved part of the *petF *ORF encoding for ferredoxin. Indeed the 5' and 3' borders of the conserved main coding region of the *petF *gene in the *T. pseudonana *chloroplast match comparable sequences in the nuclear *PETF *gene of *T. oceanica *(Figure 2 center). The situation at the 3' end illustrates that only a few changes are needed to transform parts of the AT-rich stem-loop terminating many chloroplast transcription units into a functional polyA-signal (C_2_) favouring termination of nuclear transcription. However, other conserved elements (C_1_, C_3_) are maintained as well and support the chloroplast origin of a *de novo *nuclear polyA-signal. For functional transcription, the 5' end of the *T. oceanica PETF *exon 2 had to acquire a splice acceptor site linking it to the exon 1, encoding for the cp transit peptide. We observe that the *T. pseudonana petF *sequence contains motifs in its coding region that may act as a functional splice acceptor site (coloured bars) in a nuclear genomic context. In summary, the *petF *sequence as present in the *T. pseudonana *cp genome already contains several sequence motifs that would permit an incorporation of the *petF *gene as a nearly functional exon into a nuclear genomic context, thereby facilitating the modular acquisition of a second exon and promoter through further rearrangements.

**Figure 3:**
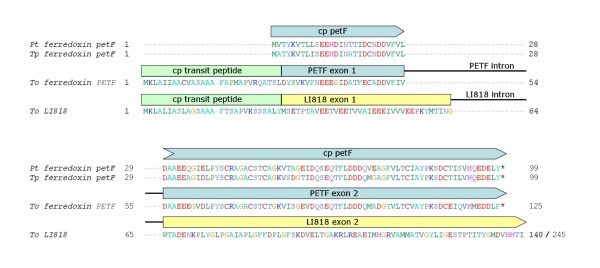
**Chloroplast Retargeting of Ferredoxin**. The *T. oceanica *nuclear ferredoxin protein sequence is aligned to its chloroplast counterparts from *T. pseudonana *and *P. tricornutum*. The ***PETF ***gene is composed of two exons, the second of which encodes the complete conserved part of the protein and is clearly of chloroplast origin, while the first exon contributes the chloroplast retargeting part and is of nuclear origin. Such a modular gene structure is not uncommon in genes encoding chloroplast targeted products as can be seen at the *T. oceanica ****LI818 ***gene belonging to the large group of nuclear ***FCP ***genes. The ***LI818 ***gene encodes a cp transit peptide in a structurally separated exon and is presented as a potential donor gene candidate to provide the freshly transferred *PETF *DNA segment with the required targeting module.

An important step towards establishing a nuclear *PETF *gene is a functional retargeting of the ferredoxin gene product for import into the chloroplast and, hence, the acquisition of a chloroplast transit peptide. In *T. oceanica *this transit peptide is encoded as a functional unit by a separate exon, suggesting the presence of a donor gene encoding for another protein of plastid destination as a potential source for such a module by duplication and exon shuffling events. An alignment of the ferredoxin gene products, showing the exon structure of the *T. oceanica PETF *and *LI818 *(a gene encoding for a chlorophyll-binding light-harvesting protein) as such a possible donor gene for the transit peptide, is provided in Figure 3. The transit peptide in diatoms [18] has a length of approx. 28 aa and, while the *T. oceanica PETF *exon 2 encodes the highly conserved part of the protein, the part encoded by the 3' half of its exon 1 is less conserved and could have evolved from a sequence similar to the *LI818 *exon 1.

### Functional expression and differential regulation of *PETF*

**Figure 4:**
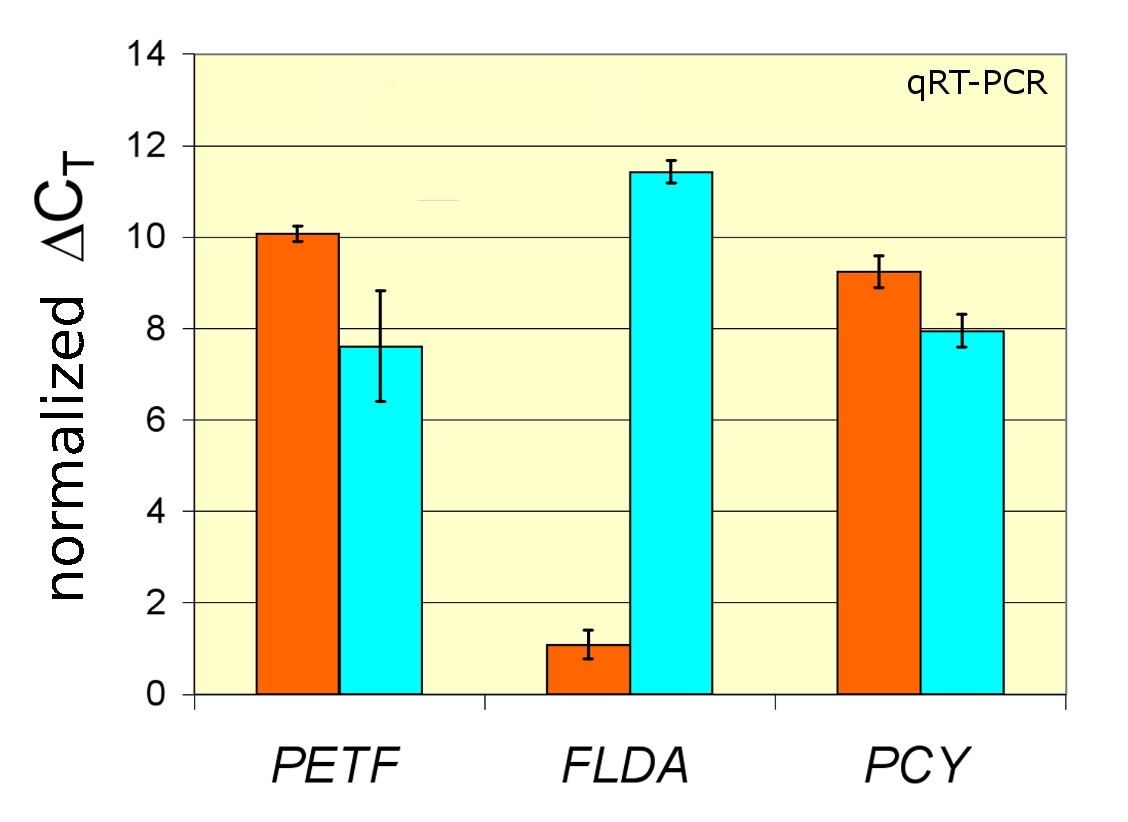
**Functional Expression and Differential Regulation of *PETF *as a Function of Iron Level**. Relative transcript abundances of the genes *FLDA*, *PETF *and *PCY *in triplicate iron-limited (light blue bars) and iron-replete cultures (orange bars) of *T. oceanica *were measured using a RT-qPCR approach with gene-specific primers pairs (Table 2). The normalized ΔC**_T _**values is calculated as 22-ΔC**_T_**, where 22 is an arbitrary number chosen to provide a comparable positive logarithmic scale for the transcript abundance of the three genes tested, where a high normalized ΔC**_T _**represents a high transcript level.

Ferredoxin contains iron and is often replaced by the iron-free flavodoxin in iron-limited growth conditions. Relative quantification studies using RT-qPCR confirm the functional expression of the transferred *T. oceanica PETF *gene. The down-regulation of *PETF *under iron-limited growth conditions was similar to that of *PCY*, the gene encoding for plastocyanin, another photosynthetic protein involved in electron transfer (Figure 4). *PETF *and *PCY *show a concerted down-regulation upon iron limitation as expected for constitutive photosynthesis genes, and in agreement with the observed down-regulation of photosynthesis in response to iron limitation. As expected, *FLDA *appears strongly up-regulated under iron-limited growth conditions, thereby supporting the idea of a mutual substitution of the ferredoxin and flavodoxin proteins.

### Novel features of the *T. oceanica *cp genome

**Figure 5:**
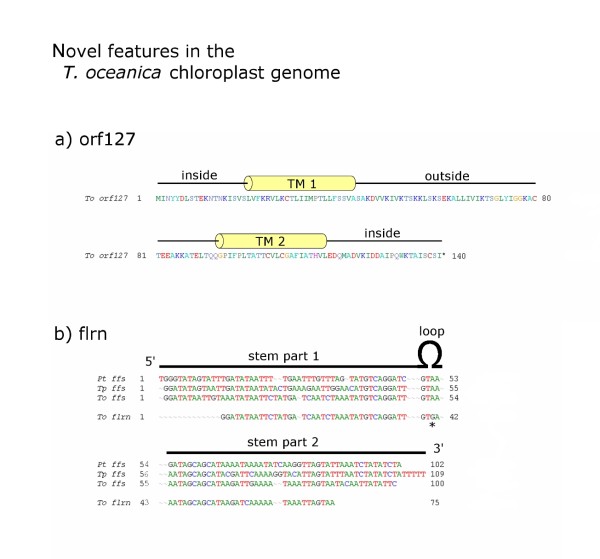
**Novel Features in the *T*. *oceanica *Chloroplast Genome**. Two novel features of the *T. oceanica *cp genome are not found in *T. pseudonana *or other diatoms. The ***orf127 ***gene encodes a hypothetical transmembrane protein of 140 aa with no homology to any other proteins (a), the RNA gene ***flrn ***appears to have originated from a duplication of the conserved ***ffs ***gene that produces the RNA component of a particle involved in membrane insertion processes. An alignment of ***flrn ***to the ***ffs ***sequences from *T. oceanica*, *T. pseudonana *and *P. tricornutum *shows the high degree of conservation (b). EST evidence for both is weak, pointing to a potential pseudogene status, though ***orf127 ***shows a well-formed genomic structure with a strong RBS (GGGAGGG) and two stem-loops that might serve as a transcriptional terminator, while the flrn gene shows a remarkable conservation compared to its ***ffs ***counterpart with one of very few mismatches placed in the loop region (*).

The genomic rearrangements in the *T. oceanica *cp genome accompanying the *petF *transfer led to a novel *orf127 *appearing at the inferred *petF *excision site. This gene encodes a protein of 140 aa containing two predicted transmembrane helices (Figure 5a) with no similarity to any known proteins in the NCBI nr database. The size and overall topology appear somewhat similar to the small transmembrane proteins from the FCP group of chlorophyll binding proteins, though sequence similarity or characteristic motifs are lacking. The gene is placed in a well-defined genomic context with an obvious ribosomal binding sequence GGGAGGG at -15 and two small inverted repeats serving as a putative rho-independent transcriptional terminator.

As a second novel feature in the *T. oceanica *chloroplast genome we identified a duplication of the *ffs *RNA gene referred to as *flrn*. The ffs RNA adopts a stem-loop structure and is part of a signal recognition particle that might play a role at insertion of proteins into the inner chloroplast membrane [19]. An alignment of the *flrn *gene with the *ffs *genes of *T. oceanica*, *T. pseudonana *and *P. tricornutum *(Figure 5b) reveals that the *flrn *gene is truncated and contains a nucleotide polymorphism in the conserved loop region. Although the similarity between *flrn *and *ffs *is high, the truncated nature of *flrn *suggests that this sequence may represent a pseudogene.

## Discussion

**Figure 6:**
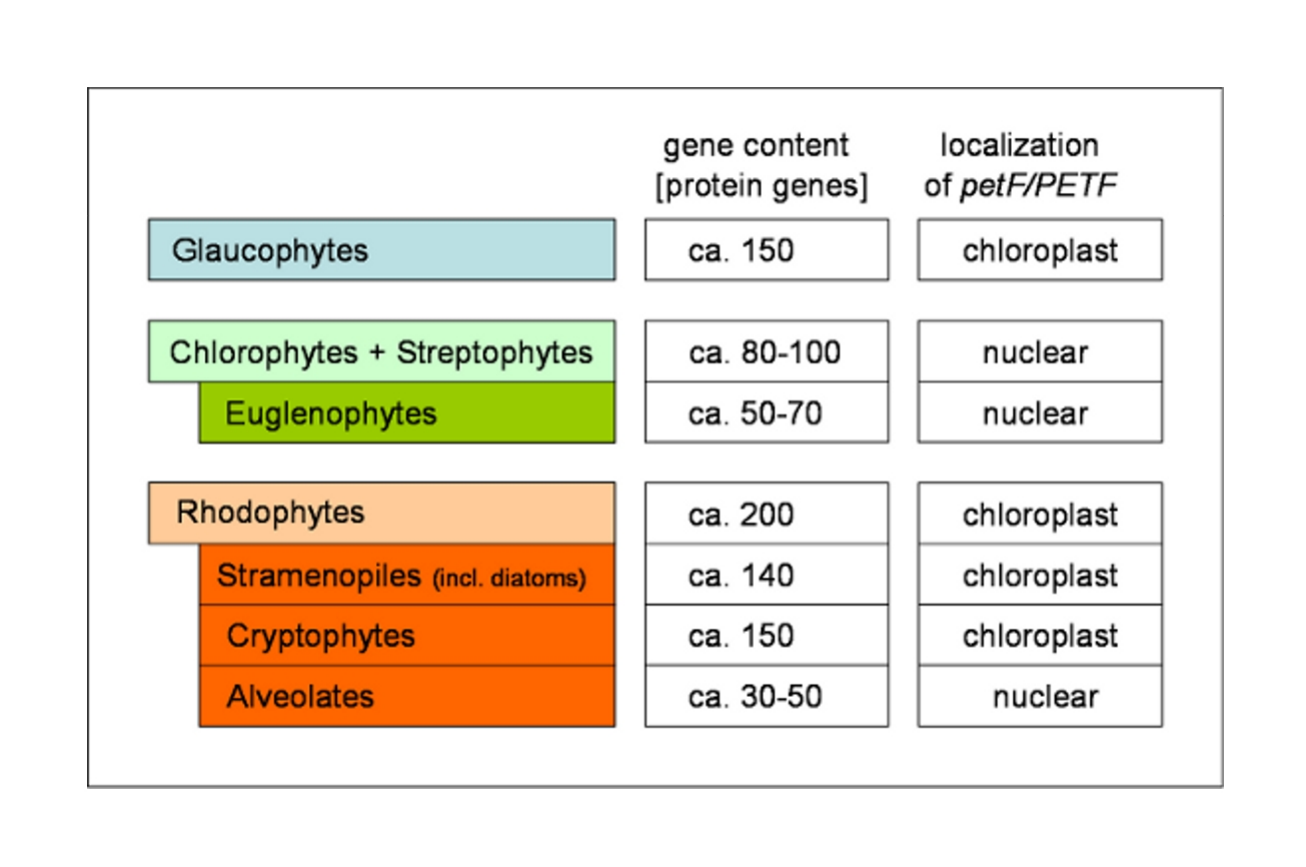
**Subcellular Localization of the *petF/PETF *Gene in the Phylogenetic Context**. A comparison of the chloroplast gene content between the main phylogenetic groups shows a reduced coding potential in the green line (Chlorophytes + Streptophytes, Euglenophytes) and in the Alveolates indicating extensive gene transfer to the nucleus or gene loss. The localization of the ferredoxin ***petF/PETF ***gene correlates with the extent of genome reduction, hence, ***petF ***is generally retained in the larger cp genomes of Glaucophytes, Rhodophytes, Stramenopiles (incl. diatoms) and Cryptophytes. Protein gene numbers are taken from the Chloroplast Genome Database [54] and represent the majority of species in the respective groups, though exceptions can deviate from the given range. Phylogenetic groups originating from secondary endosymbiosis are indented.

With new sequencing technologies emerging and an enormous increase of available sequence data, chloroplast genomes have drawn considerable interest not only for the purpose of phylogenetic reconstructions [20], but also for elucidating basic principles of genome organisation and dynamics of structural recombination events in comparative approaches [10]. Chloroplast genomes show a remarkable diversity among phototrophic eukaryotes [21]. However, their coding potential, ranging between 50 and 200 genes (Figure 6), is not even closely approaching the size and complexity of the plastid proteome needed to carry out the metabolic and physiological functions of the chloroplast. The plastid proteome is estimated to contain more than 700 proteins, many of uncertain structure and function [6]. Regulation of photosynthesis might even affect more than 4000 genes in *Arabidopsis *and novel proteins involved in elemental steps of photosynthesis are still being discovered [22].

The chloroplast genome is a dynamic structure in which the collinear binding of the inverted repeat regions likely leads to a handle-like structure isolating the LSC and SSC regions in separate domains and serving as a basis for occasional recombinational events that result in an inversion of the SSC region [23]. The weak conservation of synteny between *T. oceanica *and *T. pseudonana *cp genomes implies high structural dynamics of the circular genomic molecule, best explained by frequent inversion events. The remarkable lack of genomic rearrangements across the borders of the single copy regions, as observed by comparison between *T. oceanica *and *T. pseudonana*, could be explained by the structural separation of the LSC and SSC in a handle-like structure and supports this structural model.

The establishment of the primary endosymbiont as a chloroplast led to the loss of many of the endosymbiont's genes or their transfer to the host's nucleus. Red algal and Glaucophyte chloroplasts retain about 200 protein coding genes, while most members of the green lineage exhibit a further genome reduction to less than 100 genes, indicating functional transfer of several essential genes to the nuclear genome. The substantial retention of coding potential in the primary plastids of the red algal lineage has been hypothesized to have an impact on the chloroplast portability during secondary endosymbiosis, preferentially facilitating further endosymbiotic events of autotrophs from the red lineage over their more reduced green counterparts [7]. Gene content of extant cp genomes derived from the red-algal lineage indicate that further gene loss or transfer proceeded after secondary endosymbiosis events, leading to a gene content of approx. 140 genes in the Stramenopiles and Cryptophytes. Stronger reductions can be found within the Apicomplexa and Dinophytes, referred to as Alveolates, perhaps because the known members of the Apicomplexa and several of the Dinophytes are non-photosynthetic organisms, and sometimes parasitic [24]. All of these subgroups are regarded as descendants of an ancient red algal endosymbiont and its host [7].

Occasional free release of cp genomic DNA upon chloroplast lysis is considered an important source of chloroplast DNA for integration into the nuclear genome. This mechanism can operate only in organisms containing multiple plastids. Indeed the higher degree of gene retention in *P. tricornutum *compared to *Thalassiosira *species might be partially explained by the presence of a single, unique chloroplast in *P. tricornutum *which would not be available for lysis in the context of the living cell. *T. oceanica *and *T. pseudonana *cells on the other hand are equipped with approx. 4 plastids each [25,26].

As for other evolutionary processes, the transfer of chloroplast-encoded genes to the nuclear genome is difficult, if not impossible, to observe. Experimental approaches with transgenic tobacco have demonstrated that plastid genes can be successfully transferred to the nuclear genome [27], but most of the evidence for the inferred transfers is derived from phylogenetic and phylogenomic arguments [20]. Why certain genes are preferentially retained or transferred is puzzling and several theories have emerged ranging from a simple economic hypothesis in favour of gene transfer [28] to the CORR (CO-location for Redox Regulation) hypothesis in favour of gene retention [29]. The ferredoxin *petF *gene is not essentially retained in the CP genome, as a nuclear location is observed in several phylogenetic groups. So far, with the exception of *T. oceanica*, the *petF *gene has been retained in large cp genomes (Figure 6) such as the ones from red algae and descendants (including diatoms), and has generally been transferred to the nucleus in groups with reduced cp genomes such as Chlorophytes and Streptophytes. Several intrinsic features of the *petF *gene, conserved in the cp genomes of the closely related species *T. pseudonana *and *T. weissflogii *[30], may have facilitated the functional transfer of *petF *from the chloroplast to the nuclear genome in *T. oceanica*. Thus, in the coastal species *T. pseudonana *and *T. weissflogii*, the *petF *gene transfer may be waiting to happen as well, given appropriate environmental selection pressures. However, at this point, the establishment of the *PETF *gene in the nuclear genome of *T. oceanica *represents an exception within the diatoms for which genome information is available so far.

It has been demonstrated that the photosynthetic architecture in *T. oceanica *is better adapted to iron-limited areas than its coastal counterpart *T. weissflogii *[5]. Tolerance to iron limitation most likely arises from a combination of several genetic adaptations that contribute to a better streamlining of the photosynthetic apparatus towards low iron requirements. The low abundance of PSI relative to PSII in *T. oceanica *is clearly important in reducing the cellular iron requirements [5]. The substitution of the iron-requiring cytochrome c_6 _by the copper-containing plastocyanin in *T. oceanica *is an additional strategy to further reduce photosynthetic iron requirements, and, hence, the iron quota of this diatom [31]. Likewise, the flavodoxin expression is up-regulated upon iron limitation in many diatom species [32] while a *s*imultaneous down-regulation of the expression of the ferredoxin *petF *gene is observed (e.g. [33]). In our study, we could confirm the expression and iron-dependent regulation of the ferredoxin *PETF*, the plastocyanin *PCY *and the flavodoxin *FLDA *gene in *T. oceanica *(Figure 4). The concerted down-regulation of *PETF *and *PCY *upon iron limitation is expected as part of the general down-regulation of the photosynthetic apparatus under iron limitation. In contrast, *FLDA *is strongly up-regulated in order to substitute ferredoxin with flavodoxin, consequently keeping intact the electron-transport interface between membrane-bound light reactions and dark reactions in the cp stroma.

The contrast in photosynthetic physiology between *T. oceanica *and its coastal relatives *T. pseudonana *and *T. weissflogii *make an attractive case to infer an adaptive significance for the transfer of *petF*, an iron-regulated cp gene, to the nuclear genome. Whether the transfer of *petF *to the nuclear genome is simply a byproduct of evolutionary trends towards chloroplast genome reduction or truly confers an ecological advantage with respect to the response to iron remains uncertain, though the observed high tolerance of *T. oceanica *to severe iron limitation relative to its close relatives *T. pseudonana *and *T. weissflogii *suggests that the latter hypothesis is worthy of further investigation.

Single gene transfers are the elemental steps of cp genome reduction and may confer benefits to single species in the context of niche adaptation. It is tempting to speculate that larger phylogenetic groups whose members share a reduced cp genome (as in the green lineage) likewise emerged from a founder species that profited from the benefits of cp genome reduction and improved nuclear control over organelle function. Centralized and synchronized regulation of cp metabolism is assumed to be a potential driving force for intracellular gene transfers. Organisms that already experienced large-scale cp genome reduction should benefit as well from an improved regulation of the transferred genes. The uniform genomic situation in larger phylogenetic groups raises the question whether such competitive advantages might even apply to these groups as a whole. A comparative evaluation of this effect between the red and green lineage is complicated by the interference with different extents of gene losses in both groups as well as the presence of distinct types of photosynthetic physiology in general. However, the terrestrial environment has been conquered exclusively by members of the green lineage, and prerequisite for this achievement might (at least partially) have been the improvement of regulatory capacities linked to cp genome reduction by large scale gene transfer. The settlement of the land represented the occupation of a new ecological niche rich in abiotic stresses of a novel type. It remains to be elucidated to which extent such specific environmental stresses exert a selective pressure favouring gene transfer events [34], ultimately leading to competitive advantage and enhanced fitness.

## Conclusions

Although the chloroplast genomes of some closely related marine phytoplankton species have been sequenced, the differences between species within a genus have been small and restricted to gene reshuffling. Our findings, reporting a traceable single gene transfer from the chloroplast to the nuclear genome, are unique so far, in part because of the availability of both plastid and nuclear genome sequences for *T. oceanica *and *T. pseudonana*. The example of *petF *shows that chloroplast and nuclear genomes are of remarkable plasticity. Whether or not the gene transfer described for *T. oceanica *confers a competitive advantage still needs to be assessed through experimental approaches. Future analyses of cp genomes from a wider range of ecologically diverse species will likely reveal other surprising patterns of cp gene content, loss and regulation, and further enhance our understanding of their impacts on the evolutionary fitness of species.

## Methods

### Strains and Cultures

*T. oceanica *Hasle [25] strain CCMP1005 was grown from an axenic clonal isolate, obtained from serial dilutions of a stock culture to extinction. *T. oceanica *cells were grown in 8 l batch cultures using iron-free f/2 nutrients [35] in ASW (artificial seawater medium [36]) supplied with 10 μM FeCl_3 _at 100 μE, 25°C and a 14/10 h light/dark cycle. Cells were harvested by filtration on 47 mm 5 μ-PC [polycarbonate]-filters, resuspended into a small volume of media, followed by centrifugation at 4°C for 10 min at 11000 rpm. Cell pellets were frozen in liquid N_2 _and stored at -80°C. Genome comparison was conducted with the genome data available at JGI and NCBI for *T. pseudonana *Hasle & Heimdal CCMP1335 [26,37] and *Phaeodactylum tricornutum *Bohlin CCAP1055/1 [38,39].

### Nucleic Acid Extraction and Sequencing

Total genomic DNA for sequencing of the *T. oceanica *genome was extracted from nutrient-replete cells using the QIAGEN DNeasy kits. The quality of nucleic acids was assessed by NanoDrop UV absorption profiles and agarose gel electrophoresis. Next generation 454 sequencing technology [40] was applied to the gDNA as follows. After mechanical shearing, specific sequencing adaptors were ligated and the genomic DNA fragments were shotgun sequenced using massively parallel pyrosequencing on a 454 gs-flx instrument (Roche, Penzberg, Germany) according to the manufacturer's protocol. The resulting libraries were sequenced on a gs-flx sequencer using the standard manufacturer's protocol.

### Cp Genome Sequence Assembly and Gap Closure

1.2 Mio flx pyrosequencing reads were assembled into contigs with the TGICL assembler using the CAP3 algorithm [41]. The quality of the resulting contigs was manually confirmed by inspection using the CLVIEW cluster viewer program. Using the local BLAST package from NCBI [42], we identified 9 contigs with high sequence coverage as elements of the cp genome. Additional information extracted from the contig ends of the *ace*-file of the original assembly, enabled the manual assembly of the cp genome to near completeness. The two remaining small gaps were targeted by PCR amplification of the contig ends and demonstrated physical continuity of the gap regions. Bridging fragments were cloned in a TOPO cloning vector and their sequences determined by Sanger sequencing.

### Sequence Analysis and Annotation

The assembled chloroplast genome sequence was analyzed by BLAST against the related *T. pseudonana *cp genome sequence, the NCBI nr (non-redundant) protein database and the NCBI CDD Conserved Domain Database. The BLAST analysis revealed few obvious artificial frameshifts within the original contigs, allowing correction of the chloroplast scaffold for single nucleotide errors placed in low complexity regions of single nucleotide repeats that generally appear to be critical in 454 data. The final chloroplast scaffold was annotated using Artemis [43] and the submission software Sequin [44]. Inverted repeats at the 3' end of genes representing putative rho-independent transcriptional terminators [45,46] were identified with the EINVERTED tool from the EMBOSS software package [47]. Ribosomal Binding Sites (RBS) were determined manually from similarity to the AGGAGGT consensus sequence [48] and close proximity to the respective translation start. The contig containing the nuclear ferredoxin gene *PETF *was assembled manually from raw genomic 454 reads using local BLAST, database sequence retrieval and BioEdit [49]. Gene modelling was done with the GENSCAN webserver [50] and confirmed by NCBI blastx against the nr protein database. The derived transcript encoded a protein with high homology to *T. pseudonana *and *P. tricornutum *petF protein orthologs.

### Circular Map Construction

The circular genomic map was constructed from the primary embl-annotation file using CGVIEW [51] as follows: The *embl*-file was converted into an xml-file with the perl-script cgview_xml_builder.pl, which is enclosed in the CGVIEW package; the xml-file containing the formatting details for the circular map was then customized manually by adding functional categorization and appropriate gene symbol shapes and dimensions. The circular map was constructed from the *xml*-file as *png*-graphic, using the CGVIEW main function. Gene names were added using the open source graphics program GIMP [52].

### RT-qPCR data

Based on the transcript sequences of the 18S-rDNA, *PETF*, *FLDA *and *PCY *genes derived from the assembly data, sets of primers were designed and optimized to detect gene specific amplicons of approx. 100 bp with uniformly high amplification efficiency (>95%, Table 2). A local BLASTN analysis of the primers against all sequences available for *T. oceanica *confirmed the specificity of the primers for their respective genes. cDNA template was prepared from 1 μg RNA by reverse transcription using the QuantiTect Rev. Transcription Kit (QIAGEN), followed by digestion of residual DNA using the included gDNA wipeout reagents. The cDNA was diluted to 0.5 ng μl^-1 ^and 2.5 ng were used per qPCR reaction run on an ABI Prism 7000 (Applied Biosystems). Cycling conditions were 2 min at 50°C (once), 2 min at 95°C (once), and 40 cycles of 95°C for 0:15 min, followed by 0:30 min at 60°C. The qPCR mixtures contained 12.5 μl SYBR qPCR SUPERMIX W/ROX (Invitrogen), 0.5 μl of 10 μM forward and reverse primer each, 6.5 μl H_2_O and 5 μl of cDNA template. Gene expression was assessed as the mean from the C_T _values of 2-4 replicate reactions at a threshold level of 0.2. Relative expression of genes with respect to the 18S-rDNA gene was calculated using the ΔC_T _method (ΔC_T_[geneX] = C_T_[gene*X*] - C_T_[18S]). Final data are presented as the mean ± s.e.m. of the ΔC_T _values from three biological replicates.

**Table 2:** Oligonucleotide Primers used for RT-qPCR Analyses

Gene	Forward Primer (5'→3')	Reverse Primer (5'→3')
***PETF***	AGGCCACCTCCCTCGACTAC	GCGGCATCGACGATGAAG

***FLDA***	CCGGCCTTTTCTACTCGACC	TTGACGTCTCCGATGTCCTTC

***PCY***	CTCCGCCCCTGCTTACG	TCCCTTGCAGACAGTGACCTT

### Nomenclature of Gene Names

The nomenclature of gene names follows the recommendations for C*hlamydomonas reinhardtii *[53]. Gene names are typed in italic and uppercase for nuclear genes (*PETF*) or italic and lower case for organelle genes (*petF*).

## Authors' contributions

ML performed the gap closure cloning, the cp genome assembly and annotation, the analysis of the *petF *gene transfer, prepared the figures and wrote a major portion of the manuscript.

ASR cultured the algae, carried out the RT-qPCR work and commented on the manuscript.

MS prepared the gDNA libraries and performed the 454 sequencing.

SS provided the sequencing technology and supply.

PR coordinated the sequencing and contributed to manuscript writing.

JLR coordinated the study, isolated the *T. oceanica *gDNA, and made major contributions to discussion and manuscript writing.

All authors read and approved the final manuscript.
